# Pathophysiological roles of the serum acylcarnitine level and acylcarnitine/free carnitine ratio in patients with cardiovascular diseases

**DOI:** 10.1016/j.ijcha.2024.101386

**Published:** 2024-03-14

**Authors:** Takafumi Nakajima, Taira Fukuda, Ikuko Shibasaki, Syotaro Obi, Masashi Sakuma, Shichiro Abe, Hirotsugu Fukuda, Shigeru Toyoda, Toshiaki Nakajima

**Affiliations:** a Department of Cardiovascular Medicine, School of Medicine, Dokkyo Medical University, Shimotsuga-gun, Tochigi, Japan; b Department of Liberal Arts and Sciences, Kanagawa University of Human Services, Yokosuka, Kanagawa, Japan; c Department of Cardiovascular Surgery, School of Medicine, Dokkyo Medical University, Shimotsuga-gun, Tochigi, Japan

**Keywords:** Carnitine, Acylcarnitine, Cardiovascular disease, Chronic kidney disease, Heart failure, Muscle wasting

## Abstract

**Introduction:**

L-carnitine exerts protective effects, such as maintaining mitochondrial functions and decreasing reactive oxygen species, while acylcarnitine (AC) is linked to the development of heart failure and atherosclerosis.

**Hypothesis:**

Serum carnitines play important pathophysiological roles in cardiovascular diseases.

**Methods:**

Pre-operative biochemical data were obtained from 117 patients (71 men, average age 69.9 years) who underwent surgery for cardiovascular diseases. Measurements included pre-operative biochemical data including estimated glomerular filtration rate (eGFR), physical functions, skeletal muscle mass index (SMI) measured by bioelectrical impedance analysis, anterior thigh muscle thickness (MTh) measured by ultrasound, and routine echocardiography. Carnitine components were measured with the enzyme cycling method. Muscle wasting was diagnosed based on the Asian Working Group for Sarcopenia criteria.

**Results:**

Plasma brain natriuretic peptide (BNP) level was correlated with serum free carnitine (FC) and AC level, and the acylcarnitine/free carnitine ratio (AC/FC). AC/FC was elevated with stage of chronic kidney disease. In multivariate analysis, log (eGFR) and log (BNP) were extracted as independent factors to define log (serum AC) (eGFR: β = 0.258, p = 0.008; BNP: β = 0.273, p = 0.011), even if corrected for age, sex and body mass index. AC/FC was negatively correlated with hand-grip strength (r = -0.387, p = 0.006), SMI (r = -0.314, p = 0.012), and anterior thigh MTh (r = -0.340, p = 0.014) in men.

**Conclusions:**

A significant association between serum AC level and AC/FC, and chronic kidney disease and heart failure exists in patients with cardiovascular diseases who have undergone cardiovascular surgery. Skeletal muscle loss and muscle wasting are also linked to the elevation of serum AC level and AC/FC.

## 1 Introduction

L-carnitine, an essential compound, plays vital roles in the body, particularly in energy metabolism [1], [2], [3]. Carnitine accumulates in skeletal muscle [4], and its primary role is to transport fatty acids (FAs) to the mitochondria, thereby contributing to the skeletal muscle energy supply [5], [6]. Also, in cardiac muscle, the primary energy source of the human heart is free FAs, which are broken down by β-oxidation and enter the tricarboxylic acid (TCA) cycle, where they ultimately convert into adenosine triphosphate (ATP). Although dysfunctional FA metabolism leads to excess production of free radicals and undesired apoptosis, L-carnitine treatment positively affects the pathological course [7], and is used in patients with carnitine deficiency on hemodialysis (HD) [7], [8]. L-carnitine also helps overcome FA oxidation defects associated with metabolic diseases [9] and improves energy metabolism linked to mitochondrial disorders in diseases such as heart failure (HF) [10]. Diagnostic methods for carnitine deficiency include the liquid chromatography-mass spectrometry (LC-MS) detection and enzymatic cycling methods. While the LC-MS detection method is essential for the definitive diagnosis of congenital metabolic disorders [11], the enzyme cycling method is an alternative method for follow-up, and in most cases, the enzyme cycling method is considered a viable alternative for the diagnosis of carnitine deficiency due to other causes [12], [13], [14].

Carnitine exists as either non-esterified, termed free carnitine (FC), or esterified, known as acylcarnitine (AC). AC is produced in mitochondria as part of FA metabolism and is dysregulated in many diseases, such as sepsis [15], cancer [16], [17] and HF [18], [19], [20]. A pathway for intracellular AC formation from FA metabolism in organelles other than mitochondria has been proposed [20], [21]. In a study of patients with HF, levels of long-chain AC metabolites were significantly higher in HF with reduced ejection fraction (EF) than in HF with preserved EF, and were inversely correlated with EF [18]. Also, a study with mechanical circulatory support showed that increased serum AC levels were independently associated with adverse clinical outcomes and decreased after long-term mechanical circulatory support [19]. Thus, increased AC might be associated with an increased risk of reduced outcomes and/or adverse clinical outcomes in patients with cardiovascular disease. Also, a decrease in the acylcarnitine/free carnitine ratio (AC/FC), a marker of carnitine deficiency suggests improved mitochondrial β-oxidation [13]. It has been recently reported that aerobic and resistance exercise training decreases AC/FC, improving mitochondrial function and even inhibiting CKD progression in patients with chronic kidney disease (CKD) not receiving HD [22]. However, many aspects of the physiological role of AC and AC/FC remain to be elucidated, especially in patients with cardiovascular disease. Therefore, this study aimed to investigate how serum AC level and AC/FC play pathological roles using the enzyme cycling method in patients with cardiovascular disease undergoing cardiovascular surgery.

## 2 Materials and methods

### 2.1 Participants

A total of 117 patients (71 men, average age 69.9 years) undergoing cardiovascular surgery between October 13th 2015 and January 31st 2019 at Dokkyo Medical University Hospital were included in this cross-sectional study. The study was approved by the Bioethics Committee of Dokkyo Medical University Hospital (No. 27074), and written informed consent was obtained from all participants.

Biochemical data were measured before surgery and analyzed by routine chemical methods in the Dokkyo Medical University Hospital clinical laboratory. Fasting total cholesterol, albumin (Alb), hemoglobin (Hb), HbA1c, brain natriuretic peptide (BNP), low-density lipoprotein-cholesterol, high density lipoprotein cholesterol, total cholesterol, triglyceride levels, and estimated glomerular filtration rate (eGFR) were measured. Levels of the inflammatory marker, high-sensitivity C-reactive protein, were measured with a latex-enhanced nephelometric immunoassay (N Latex CRP II and N Latex SAA, Dade Behring Ltd., Tokyo, Japan).

To measure carnitine components and cytokine concentrations, peripheral venous blood was collected into pyrogen-free tubes with and without ethylenediaminetetraacetic acid (EDTA) on the morning of surgery. For plasma, the EDTA-containing tubes were placed on melting ice, subsequently centrifuged at 1500*g* for 20 min, 10 min of which was at 4 °C. Plasma and serum were stored in aliquots at −80 °C for all enzyme-linked immunosorbent assays (ELISAs). Serum tumor necrosis factor (TNF)-α level was measured using a Human TNF-α ELISA Kit (Quantikine® HS ELISA, R&D Systems, Inc., Minneapolis, MN, USA). The detection threshold for TNF-α was 0.022 pg/mL. Serum growth differentiation factor (GDF)-15 level was measured using a Human GDF-15 ELISA Kit (Quantikine® DGD150 for GDF-15, R&D Systems, Inc.). The detection threshold for GDF-15 was 0.002 ng/mL. Serum fibroblast growth factor (FGF)-21 level was measured using a Human FGF-21 ELISA Kit (Quantikine® ELISA, R&D Systems, Inc.). The detection threshold for FGF-21 was 4.67 pg/mL. Serum carnitine levels were measured with enzymatic cycling. Carnitine insufficiency was defined as a reduced FC level or an increased AC/FC, which indicates the presence of abnormal carnitine metabolism, reflecting relative carnitine deficiency due to increased demand of fatty acid metabolism. In accordance with previous studies, the diagnostic criterion for carnitine deficiency in this study was a low FC level (<20 μmol/L) or a high AC/FC (>0.4) [23], [24].

### 2.2 Transthoracic echocardiography

Each patient underwent preoperative transthoracic echocardiography. Two-dimensional (2D) images were recorded with an iE33 and EPICQ7 cardiovascular ultrasound system (PHILIP, Amsterdam, Netherlands) with a 1.7–3.4 MHz Doppler transducer. 2D echocardiography was performed according to the recommendations of the American Society of Echocardiography. Left atrial diameter (LAD), left ventricular end-diastolic diameter (LVDd), left ventricular end-systolic diameter (LVDs), interventricular septal thickness (IVSth), and left ventricular posterior wall thickness (PWth) were measured using the parasternal long-axis view. Left ventricular mass (LV mass) was estimated by LVDd and wall thickness (IVSth and PWth) and then indexed to body surface area.

LVmass = 0.8{1.04[(LVDd + IVSth + PWth)^3^ − LVDd^3^]} + 0.6

Left ventricular end-diastolic volume (LVEDV) and end-systolic volume (LVESV) were measured from the apical view with the biplane method. Left ventricular ejection fraction (LVEF) was calculated using the Simpson method.

LVEF = 100(LVEDV − LVESV)/LVEDV

Doppler echocardiography was performed and E/e’ was determined by the ratio of early-diastolic left ventricular inflow velocity (E) to early-diastolic mitral annular velocity (e’).

### 2.3 Measurement of hand-grip strength, knee extension voluntary isometric contraction, and walking speed

Maximum voluntary isometric contraction (MVIC) of the hand grip was determined with a factory-calibrated hand dynamometer (TKK 5401, TAKEI Scientific Instruments Co., Ltd., Tokyo, Japan). Each subject underwent two trials, and the highest value of the two trials was used for analysis. MVIC of the knee extensors was determined with a digital handheld dynamometer (μTas MT-1, ANIMA Co., Ltd., Tokyo, Japan) as described previously [25], [26], [27]. Measurements were conducted with participants in the sitting position in which the trunk was vertical and the hip and knee joints were bent to 90 degrees, and the upper limbs were located in front of the anterior chest. The position of the sensor was set at the distal end of the lower leg, and a fixing belt was attached at a right angle to the direction in which the force was applied. Each subject performed two trials with an interval of at least 2 min between trials. The maximum value was adopted as the muscle strength of isometric knee extension. The walking speed was calculated by measuring the time required to walk 4 m at a typical pace.

### 2.4 Measurements with the bioelectrical impedance analyzer

A multi-frequency bioelectrical impedance analyzer (BIA), InBody S10 Biospace device (Biospace Co, Ltd, Korea/Model JMW140) was used according to the manufacturer’s guidelines, as described previously [25], [26], [27]. Thirty impedance measurements were obtained at 6 different frequencies (1, 5, 50, 250, 500, and 1000 kHz) at 5 segments of the body (right and left arms, trunk, and right and left legs). The measurements were carried out while the subjects rested quietly in the supine position, with their elbows extended and relaxed along their trunk. Body fat volume, body fat percentage, and skeletal muscle volume were measured. Skeletal muscle mass index (SMI; appendicular skeletal muscle mass/height^2^, kg/m^2^) was measured as the sum of lean soft tissue of the two upper limbs and two lower limbs. In this study, muscle wasting was defined according to the Asian Working Group for Sarcopenia (AWGS) criteria (hand-grip strength < 26 kg for men and < 18 kg for women or gait speed ≤ 0.8 m/sec, and SMI < 7.0 kg/m^2^ for men and < 5.7 kg/m^2^ for women) [28].

### 2.5 Measurement of muscle thickness by ultrasound

Quadriceps muscle thickness was measured by ultrasound evaluation at the midpoint of the thigh length with a real-time linear electronic scanner with a 10.0-MHz scanning head and ultrasound probe (L4-12 t-RS Probe, GE Healthcare, Tokyo, Japan) and LOGIQ e ultrasound (GE Healthcare, Tokyo, Japan) as previously described [25], [26], [27]. The scanning head was coated with a water-soluble transmission gel to provide acoustic contact without depressing the dermal surface. The subcutaneous adipose tissue-muscle interface and the muscle-bone interface were identified from the ultrasound image. The perpendicular distance from the adipose tissue-muscle interface to the muscle-bone interface was considered to represent the quadriceps muscle thickness. The anterior mid-thigh muscle thickness (MTh) was measured in the supine position; the measurement was performed twice at each side of the thigh, and the average value was adopted.

### 2.6 Statistical analysis

Data are presented as mean ± standard deviation (SD), or number (proportion). After testing for normality (Kolmogorov-Smirnov), the comparison of means between groups was analyzed with a two-sided, unpaired Student’s *t*-test in the case of normally distributed parameters or with the Mann-Whitney-*U* Test in the case of non-normally distributed parameters. Associations among parameters were evaluated with Pearson or Spearman correlation coefficients. Multiple linear regression analysis with serum AC or AC/FC as the dependent variable was performed to identify independent predictors (clinical laboratory data). Also, multiple linear regression analysis with eGFR or BNP as the dependent variable was performed to identify independent predictors (serum AC or AC/FC). When the residuals of the dependent or independent data were not normally distributed, they were logarithmically transformed to achieve a normal distribution. Age, sex, and body mass index (BMI) were employed as covariates. All analyses were performed with SPSS version 24 (IBM Corp., New York, USA) for Windows. A p value of < 0.05 was considered significant.

## 3 Results

### 3.1 Patients

The clinical characteristics of the study patients are shown in Table 1. Their BMI was 23.6 ± 3.8 kg/m^2^ and preoperative New York Heart Association functional classification was 2.2 ± 1.0. The majority of patients exhibited conventional risk factors, such as hypertension, diabetes, dyslipidemia, current smoking, and HD. Patients underwent coronary artery bypass grafting (CABG) (n = 21 [18 %]), aortic valve replacement (AVR)/transcatheter aortic valve implantation (TAVI) (n = 28 [24 %]), mitral valve replacement (MVR)/mitral valve plasty (MVP) with or without tricuspid valve replacement (TVR)/tricuspid annuloplasty (TAP) (n = 25 [21 %]), CABG combined with valve replacement or repair (n = 10 [9 %]), AVR combined with other valve or aortic diseases (n = 15 [13 %]), aortic diseases (n = 9 [8 %]), or other procedures (n = 9 [8 %]). All patients were receiving pharmacologic treatment, including β-blocking agents (n = 57 [49 %]), calcium-channel blockers (n = 39 [33 %]), angiotensin receptor blockers/angiotensin converting enzyme inhibitors (n = 66 [56 %]), diuretics (n = 55 [47 %]), statins (n = 57 [49 %]), and anti-diabetic drugs (n = 25 [21 %]). Total carnitine (TC) and FC concentrations were 66.8 ± 32.6 μmol/L and 50.3 ± 23.5 μmol/L, respectively. AC concentration was 16.5 ± 10.7 μmol/L and AC/FC was 0.34 ± 0.14. The difference in serum carnitine components (TC, FC, AC, AC/FC) between men and women was not significant. Carnitine deficiency was observed in 6 (5 %) and 29 (25 %) of 117 patients according to low FC level and high AC/FC, respectively.

**Table 1:** Patient Characteristics.

	Total patients		
Number	117	eGFR, mL/min/1.73 m^2^	58.9 ± 26.2
Male / Female	71 (61 %)/ 46 (39 %)	BNP, pg/mL	381 ± 582
Age, y	69.9 ± 12.7	Creatinine, mg/dL	1.6 ± 2.3
BMI, kg/m^2^	23.6 ± 3.8	HbA1c, %	6.1 ± 0.8
Atrial fibrillation	36 (31 %)	LDL cholesterol, mg/dL	92 ± 28
NYHA functional classification	2.2 ± 1.0	HDL cholesterol, mg/dL	54 ± 17
Risk factors		Total cholesterol, mg/dL	168 ± 38
Hypertension	83 (71 %)	TG, mg/dL	107 ± 63
Diabetes	27 (23 %)	hsCRP, mg/L	0.8 ± 1.7
Dyslipidemia	57 (49 %)	Carnitine and derivatives	
Smoking	19 (16 %)	TC, μmol/L	66.8 ± 32.6
Hemodialysis	9 (8 %)	FC, μmol/L	50.3 ± 23.5
Cardiovascular surgery		AC, μmol/L	16.5 ± 10.7
CABG	21 (18 %)	AC/FC	0.34 ± 0.14
AVR or TAVI	28 (24 %)	Echocardiographic findings	
MVR (MVP) with or without TVR (TAP)	25 (21 %)	LAD, mm	44.0 ± 10.4
CABG combined with valve replacement / repair (AVR, MVP, TAP)	10 (9 %)	LVDd, mm	51.9 ± 10.5
AVR combined with other valve (MVP, TAP) or aortic diseases (TAR)	15 (13 %)	LVDs, mm	36.0 ± 9.8
Aortic diseases (TAR, TEVAR, et al.)	9 (8 %)	EF, %	57.7 ± 12.6
Others	9 (8 %)	LVMI, g/m^2^	113 ± 39
Concomitant medications		E/e’	20.3 ± 11.2
β-blockers	57 (49 %)		
Ca-blockers	39 (33 %)		
ACE-Is/ARBs	66 (56 %)		
Diuretics	55 (47 %)		
Statins	57 (49 %)		
Anti-diabetic drugs	25 (21 %)		

Data are shown as mean ± SD, or number (proportion). BMI, body mass index; NYHA, New York Heart Association; CABG, coronary artery bypass grafting; AVR, aortic valve replacement; SD, standard deviation; TAVI, transcatheter aortic valve implantation; MVR, mitral valve replacement; MVP, mitral valve plasty; TVR, tricuspid valve replacement; TAP, tricuspid annuloplasty; TAR, total arch replacement; TEVAR, thoracic endovascular aortic repair; ACEI, angiotensin converting enzyme inhibitor; ARB, angiotensin II receptor blocker; Anti-diabetic drugs (i.e., α-glucosidase inhibitor, sulfonylurea, biguanide, dipeptidyl peptidase-4 inhibitor, sodium glucose cotransporter 2 inhibitor); eGFR, estimated glomerular filtration rate; BNP, brain natriuretic peptide; HbA1c, hemoglobin A1c; LDL, low-density lipoprotein; HDL, high-density lipoprotein; TG, triglyceride; hsCRP, high-sensitivity C-reactive protein; TC, total carnitine; FC, free carnitine; AC, acylcarnitine; AC/FC, the acylcarnitine/free carnitine ratio; LAD, left atrial diameter; LVDd, left ventricular end-diastolic diameter; LVDs, left ventricular end-systolic diameter; EF, ejection fraction; LVMI, left ventricular mass index; E/e’, the ratio of early diastolic mitral inflow velocity to early diastolic mitral annular velocity.

### 3.2 Correlations between serum carnitine components and clinical data

The correlations between serum carnitine components and clinical data are shown in Table 2. The serum carnitine components (TC, FC, AC, AC/FC) were not correlated with age, sex, or BMI. The concentrations of AC (r = -0.360, p < 0.001) and the AC/FC (r = -0.543, p < 0.001) were negatively correlated with eGFR, as shown in Fig. 1A. AC/FC was negatively correlated with Hb (r = -0.257, p = 0.004) and Alb (r = -0.281, p = 0.002). The serum TC and FC concentrations were positively correlated with BNP (TC: r = 0.210, p = 0.019; FC: r = 0.177, p = 0.048), as shown in Fig. 1B. The serum AC concentration and the AC/FC were also positively correlated with BNP (AC: r = 0.281, p = 0.001; AC/FC: r = 0.257, p = 0.004). Table 2 also shows the relationships between carnitine components. The serum FC concentration was positively correlated with the TC (r = 0.962, p < 0.001) and AC concentrations (r = 0.574, p < 0.001) and negatively correlated with the AC/FC (r = -0.202, p = 0.023). The AC concentration was positively correlated with the AC/FC (r = 0.582, p < 0.001).

**Table 2:** Relationship between carnitine components and clinical data.

	TC	FC	AC	AC/FC
	r-value (p-value)	r-value (p-value)	r-value (p-value)	r-value (p-value)
Age	−0.011 (0.904)	−0.034 (0.718)	0.051 (0.588)	0.139 (0.134)
Sex	−0.116 (0.212)	−0.099 (0.287)	−0.133 (0.153)	−0.104 (0.265)
BMI	0.034 (0.711)	0.043 (0.642)	0.040 (0.661)	−0.033 (0.715)
eGFR	−0.112 (0.217)	−0.024 (0.787)	−0.360 (<0.001)***	−0.543 (<0.001)***
Hb	0.054 (0.552)	0.089 (0.325)	−0.120 (0.183)	−0.257 (0.004)**
LDL cholesterol	0.026 (0.782)	0.036 (0.697)	0.003 (0.972)	−0.017 (0.851)
HDL cholesterol	−0.172 (0.058)	−0.127 (0.163)	−0.234 (0.010)*	−0.070 (0.447)
Total cholesterol	−0.021 (0.816)	0.011 (0.908)	−0.084 (0.359)	0.139 (0.125)
Alb	0.066 (0.470)	0.115 (0.205)	−0.093 (0.305)	−0.281 (0.002)**
CRP	−0.017 (0.855)	−0.024 (0.795)	0.031 (0.734)	0.098 (0.279)
Creatinine	0.139 (0.122)	0.063 (0.486)	0.343 (<0.001)***	0.493 (<0.001)***
HbA1C	−0.021 (0.823)	−0.050 (0.586)	0.084 (0.358)	0.060 (0.514)
BNP	0.210 (0.019)*	0.177 (0.048)*	0.281 (0.001)**	0.257 (0.004)**
TC	–	0.962 (<0.001)***	0.755 (<0.001)***	0.022 (0.811)
FC	–	–	0.574 (<0.001)***	−0.202 (0.023)*
AC	–	–	–	0.582 (<0.001)***

* p < 0.05, ** p < 0.01, *** p < 0.001. TC, total carnitine; FC, free carnitine; AC, acylcarnitine; AC/FC, the acylcarnitine/free carnitine ratio; BMI, body mass index; eGFR, estimated glomerular filtration rate; Hb, hemoglobin; LDL, low-density lipoprotein; HDL, high-density lipoprotein; Alb, albumin; CRP, C-reactive protein; HbA1c, hemoglobin A1c; BNP, brain natriuretic peptide.

**Fig. 1A:**
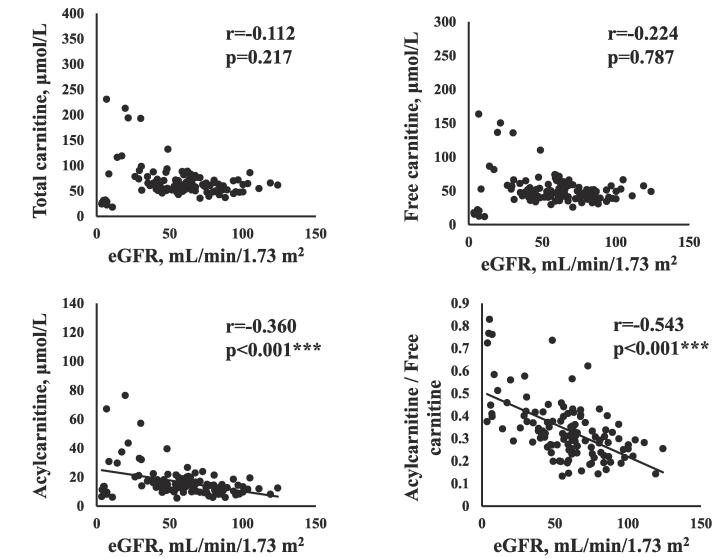
Relationship between estimated glomerular filtration rate and carnitine components.

**Fig. 1B:**
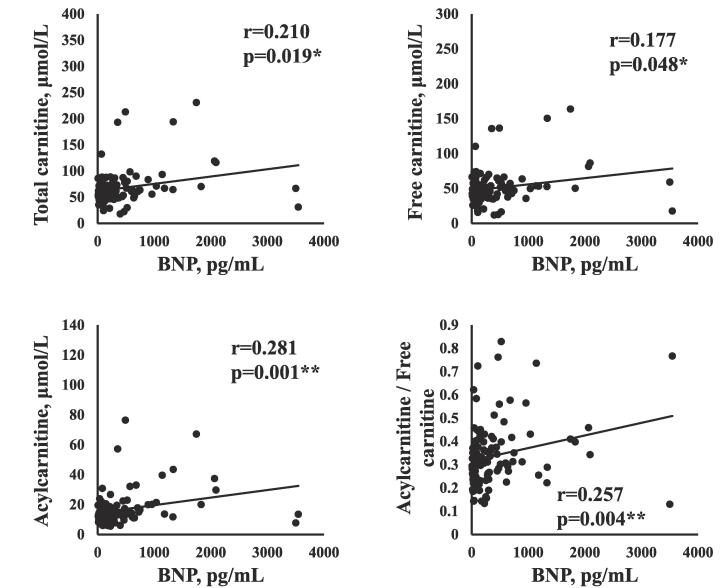
Relationship between plasma brain natriuretic peptide and carnitine components.

### 3.3 CKD stage and clinical data

The mean eGFR of all patients was 58.9 ± 26.2 mL/min/1.73 m^2^. Patients were also classified into three groups based on eGFR level (Table 3): Group 1, normal (eGFR ≥ 90 mL/min/1.73 m^2^) and low (eGFR 60–89 mL/min/1.73 m^2^); Group 2, moderate (eGFR 30–59 mL/min/1.73 m^2^); Group 3, severe (eGFR 15–29 mL/min/1.73 m^2^) and kidney failure (eGFR < 15 mL/min/1.73 m^2^). eGFR and Alb were decreased depending on the stage of CKD. The AC/FC was elevated depending on the stage of CKD (Group 1: 0.28 ± 0.09, Group 2: 0.32 ± 0.10, Group 3: 0.51 ± 0.16). In contrast, TC, FC, and AC concentrations were not significantly different among these groups. TNF-α, GDF-15, and FGF-21 were elevated depending on the stage of CKD.

**Table 3:** CKD stage and serum data.

	Group 1 (eGFR ≥ 60 mL/min/1.73 m^2^)	Group 2 (eGFR 30–59 mL/min/1.73 m^2^)	Group 3 (eGFR < 30 mL/min/1.73 m^2^)
Age, y	67.3 (14.36)	71.83 (10.6)	71.6 (7.96)
BMI, kg/m^2^	22.98 (3.72)	25.05 (4.53)	22.95 (2.68) /*
eGFR, mL/min/1.73 m^2^	81.88 (28.6)	48.0 (8.75)***	12.03 (8.76)***/***
BNP, pg/mL	307.88 (510.9)	290.89 (328.5)	847.9 (945.5)**
Hb, g/dL	12.78 (2.00)	12.09 (1.90)	10.87 (1.36)***
Alb, g/dL	3.93 (0.60)	3.85 (0.58)**	3.42 (0.56)**
TC, μmol/L	60.83 (12.56)	69.08 (26.54)	73.81 (59.1)
FC, μmol/L	47.41 (10.23)	52.24 (19.80)	57.28 (49.09)
AC, μmol/L	13.41 (4.51)	16.84 (8.72)	25.76 (20.22)
AC/FC	0.28 (0.09)	0.32 (0.10)***	0.51 (0.16)***
TNF-α, pg/mL	0.98 (0.51)	1.17 (0.40)***	2.21 (0.78)***
GDF-15, pg/mL	1067.0 (711.6)	2072.9 (1974.8)***	5116.6 (2425.8)***/***
FGF-21, pg/mL	226.3 (228.7)	339.2 (394.7)***	1497.7 (1411.8)***

Data are shown as mean (SD). * p < 0.05, ** p < 0.01, *** p < 0.001 vs. Group1/Group 2. CKD, chronic kidney disease; BMI, body mass index; eGFR, estimated glomerular filtration rate; BNP, brain natriuretic peptide; Hb, hemoglobin; Alb, albumin; TC, total carnitine; FC, free carnitine; AC, acylcarnitine; AC/FC, the acylcarnitine/free carnitine ratio; TNF, tumor necrosis factor; GDF, growth differentiation factor; FGF, fibroblast growth factor.

### 3.4 Correlations between concentrations of serum carnitine components, physical function and BIA findings

**Fig. 2:**
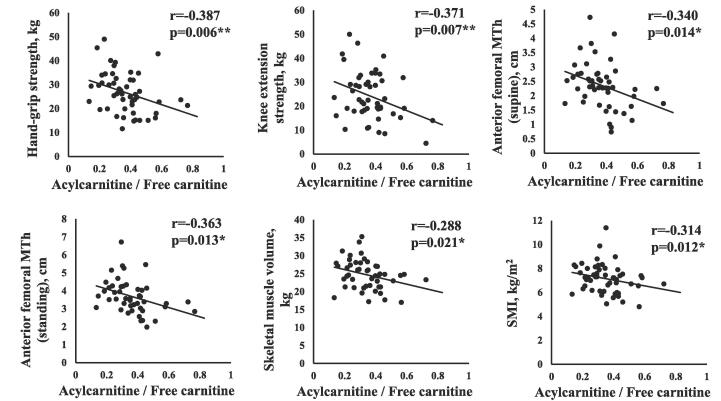
Relationship between serum carnitine components and physical data in male patients.

Tables 4A and 4B show the relationships between serum carnitine components (TC, FC, AC, AC/FC), physical function, and BIA findings in total patients and in male patients, respectively. The serum AC level was negatively correlated with hand-grip strength and anterior thigh MTh in men. The AC/FC was negatively correlated with hand-grip strength (r = -0.387, p = 0.006), SMI (r = -0.314, p = 0.012), and anterior thigh MTh (r = -0.340, p = 0.014) in men, as shown in Fig. 2.

**Table 4:** Correlations between serum carnitine components concentrations, physical function and the BIA findings.

A. Total patients				
	TC	FC	AC	AC/FC
	r-value (p-value)	r-value (p-value)	r-value (p-value)	r-value (p-value)
Hand-grip strength	0.020 (0.848)	0.060 (0.569)	−0.065 (0.542)	−0.155 (0.141)
Knee extension	−0.016 (0.883)	0.025 (0.819)	−0.098 (0.370)	−0.194 (0.073)
Anterior thigh MTh (supine)	−0.103 (0.349)	−0.045 (0.681)	−0.207 (0.056)	−0.202 (0.062)
Anterior thigh MTh (standing)	−0.144 (0.211)	−0.077 (0.504)	−0.271 (0.017)*	−0.250 (0.028)*
Walking speed	0.049 (0.646)	0.064 (0.550)	0.015 (0.889)	−0.154 (0.148)
Skeletal muscle volume	0.112 (0.262)	0.152 (0.127)	0.014 (0.888)	−0.152 (0.127)
SMI	0.040 (0.691)	0.090 (0.373)	−0.068 (0.503)	−0.196 (0.050)
Lean body mass	0.118 (0.237)	0.154 (0.123)	0.029 (0.776)	−0.132 (0.186)
Body fat mass	0.032 (0.749)	0.040 (0.688)	0.043 (0.671)	−0.068 (0.496)
Body fat percentage	−0.051 (0.609)	−0.043 (0.668)	−0.061 (0.542)	−0.009 (0.928)
Muscle volume (lower extremities)	0.110 (0.272)	0.154 (0.123)	0.004 (0.969)	−0.163 (0.101)

B. Male Patients				
	TC	FC	AC	AC/FC
	r-value (p-value)	r-value (p-value)	r-value (p-value)	r-value (p-value)
Hand-grip strength	−0.159 (0.246)	−0.090 (0.512)	−0.283 (0.036)*	−0.387 (0.006)**
Knee extension	−0.171 (0.229)	−0.116 (0.417)	−0.267 (0.058)	−0.371 (0.007)**
Anterior thigh MTh (supine)	−0.184 (0.191)	−0.126 (0.374)	−0.286 (0.040)*	−0.340 (0.014)*
Anterior thigh MTh (standing)	−0.219 (0.144)	−0.154 (0.307)	−0.343 (0.020)*	−0.363 (0.013)*
Walking speed	0.033 (0.813)	0.040 (0.775)	0.016 (0.907)	−0.109 (0.434)
Skeletal muscle volume	−0.060 (0.636)	−0.010 (0.938)	−0.154 (0.223)	−0.288 (0.021)*
SMI	−0.136 (0.287)	−0.079 (0.540)	−0.237 (0.062)	−0.314 (0.012)*
Lean body mass	−0.054 (0.674)	−0.004 (0.976)	−0.147 (0.245)	−0.259 (0.039)*
Body fat mass	0.003 (0.979)	0.021 (0.867)	−0.033 (0.799)	−0.116 (0.359)
Body fat percentage	0.051 (0.979)	0.059 (0.645)	0.030 (0.817)	−0.076 (0.550)

* p < 0.05, ** p < 0.01. TC, total carnitine; FC, free carnitine; AC, acylcarnitine; AC/FC, the acylcarnitine/free carnitine ratio; MTh, muscle thickness; SMI, skeletal muscle mass index.

### 3.5 Correlations between levels of serum carnitine components and cytokines

Table 5 shows the relationships between cytokines and carnitine components. Both the serum AC concentration and the AC/FC were positively correlated with serum TNF-α, GDF-15, and FGF-21 levels, whereas the serum TC concentration was positively correlated only with the serum TNF-α concentration, and the serum FC concentration did not correlate with the level of any of the cytokines.

**Table 5:** Relationships between cytokines and carnitine components.

	TNF-α	GDF-15	FGF-21
	r-value (p-value)	r-value (p-value)	r-value (p-value)
AC	0.307 (<0.001)***	0.196 (0.027)*	0.266 (0.002)**
AC/FC	0.420 (<0.001)***	0.494 (<0.001)***	0.372 (<0.001)***
TC	0.179 (0.044)*	0.125 (0.160)	0.096 (0.282)
FC	0.143 (0.109)	0.045 (0.614)	0.036 (0.690)

*p < 0.05, ** p < 0.01, *** p < 0.001. TNF, tumor necrosis factor; GDF, growth differentiation factor; FGF, fibroblast growth factor; AC, acylcarnitine; AC/FC, the acylcarnitine/free carnitine ratio; TC, total carnitine; FC, free carnitine.

### 3.6 Correlations between levels of serum carnitine components and echocardiographic findings

Table 6 shows the relationships between serum AC, AC/FC, and echocardiographic findings in men and women. The baseline echocardiographic data are shown in Table 1. The serum AC concentration was correlated with the LAD (r = 0.252, p = 0.041) and LVDs (r = 0.248, p = 0.043) in men. It was also negatively correlated with the LVEF (r = -0.286, p = 0.033) in men.

**Table 6:** Relationship between carnitine components and echocardiographic findings.

	LAD	LVDd	LVDs	EF (bp) %	LVMI	E/e’
AC (men)	**0.252** (0.041)*	0.210 (0.088)	**0.248** (0.043)*	**−0.286** (0.033)*	0.070 (0.577)	0.055 (0.673)
AC (women)	0.044 (0.771)	0.070 (0.642)	−0.035 (0.818)	0.134 (0.411)	−0.165 (0.283)	0.063 (0.684)
AC/FC (men)	0.137 (0.272)	0.069 (0.578)	0.101 (0.415)	−0.191 (0.158)	0.012 (0.923)	**0.293** (0.021)*
AC/FC (women)	−0.010 (0.946)	0.044 (0.771)	−0.091 (0.547)	0.154 (0.342)	−0.222 (0.148)	0.101 (0.514)

*p < 0.05. AC, acylcarnitine; AC/FC, the acylcarnitine/free carnitine ratio; LAD, left atrial diameter; LVDd, left ventricular end-diastolic diameter; LVDs, left ventricular end-systolic diameter; EF, ejection fraction; bp, biplane; LVMI, left ventricular mass index; E/e’, the ratio of early diastolic mitral inflow velocity to early diastolic mitral annular velocity.

### 3.7 Multiple linear regression analysis of serum carnitine components and clinical data

Linear regression analysis of serum AC concentrations and AC/FC with clinical data was investigated in all patients. Multiple regression analysis showed that log (BNP) (β = 0.273, p = 0.011) and log (eGFR) (β = 0.258, p = 0.008) were independent predictors of log (serum AC) after adjusting for age, sex, and BMI (Table 7A). Also, log (eGFR) (β = -0.519, p < 0.001) was an independent predictor of log (AC/FC) after adjusting for age, sex, and BMI. Multiple regression analysis showed that log (AC/FC) (β = -0.628, p < 0.001) was an independent predictor of log (eGFR) after adjusting for age and sex (Table 7B). Also, log (serum AC) (β = 0.262, p = 0.011) was an independent predictor of log (BNP) after adjusting for age and sex.

**Table 7:** Multiple linear regression analysis of serum AC, AC/FC, and clinical data A. Multiple linear regression analysis of serum AC, AC/FC, and clinical data.

**A. Multiple linear regression analysis of serum AC, AC/FC, and clinical data**				
		Dependent variable: serum AC (log)		
	Model 1	Model 2	Model 3	Model 4

Independent variable	β-value (p-value)	β-value (p-value)	β-value (p-value)	β-value (p-value)
eGFR (log)	-0.267 (0.005)**	-0.280 (0.003)**	-0.267 (0.011)*	0.258 (0.008)**
Hb	0.002 (0.857)	-0.053 (0.643)	-0.082 (0.480)	-0.085 (0.468)
Alb	-0.021 (0.378)	0.003 (0.976)	0.043 (0.722)	0.040 (0.741)
BNP (log)	0.221 (0.029)*	0.260 (0.012)*	0.262 (0.011)*	0.273 (0.011)*
		Dependent variable: AC/FC (log)		

	Model 1	Model 2	Model 3	Model 4
Independent variable	β-value (p-value)	β-value (p-value)	β-value (p-value)	β-value (p-value)

eGFR (log)	-0.510 (<0.001)***	-0.514 (<0.001)***	-0.516 (<0.001)***	-0.519 (<0.001)***
Hb	-0.038( (0.696)	-0.056 (0.583)	-0.052 (0.619)	-0.051 (0.627)
Alb	-0.175 (0.086)	-0.167 (0.105)	-0.171 (0.113)	-0.171 (0.117)
BNP (log)	-0.009 (0.915)	0.003 (0.551)	0.003 (0.973)	0.000 (0.996)
Model 1, unadjusted; Model 2, adjusted by age, Model 3, adjusted by age and sex, Model 4, adjusted by age, sex, and BMI.				

**B. Multiple linear regression analysis of eGFR, BNP and serum AC, AC/FC**			
		Dependent variable: eGFR (log)	
	Model 1	Model 2	Model 3

Independent variable	β-value (p-value)	β-value (p-value)	β-value (p-value)
Serum AC (log)	0.119 (0.173)	0.114 (0.193)	0.133 (0.133)
AC/FC (log)	-0.646 (<0.001)***	-0.632 (<0.001)***	-0.628 (<0.001)***

		Dependent variable: BNP (log)	

	Model 1	Model 2	Model 3

Independent variable	β-value (p-value)	β-value (p-value)	β-value (p-value)
Serum AC (log)	0.248 (0.017)*	0.263 (0.010)*	0.262 (0.011)*
AC/FC (log)	0.099 (0.334)	0.047 (0.467)	0.047 (0.645)

Model 1, unadjusted; Model 2, adjusted for age, Model 3, adjusted for age and sex.

* p < 0.05, ** p < 0.01, *** p < 0.001. AC, acylcarnitine; AC/FC, the acylcarnitine/free carnitine ratio; eGFR, estimated glomerular filtration rate; Hb, hemoglobin; Alb, albumin; BNP, brain natriuretic peptide.

### 3.8 Clinical differences between patients with and without muscle wasting

Muscle wasting was identified in 36 (36 %) of 99 patients based on the AWGS criteria. Table 8 shows a comparison of various parameters of patients with and without muscle wasting. Compared to men and women without muscle wasting, those with muscle wasting had a significantly higher mean age. Men and women with muscle wasting had significantly higher BNP levels compared to those without muscle wasting. BMI and eGFR were significantly lower in men with muscle wasting compared to those without muscle wasting, and tended to be lower in women. The serum AC concentration and AC/FC in men with muscle wasting were higher than those without muscle wasting, and tended to be higher in women. In contrast, the serum concentrations of TC and FC were not significantly different between patients with and without muscle wasting.

**Table 8:** Clinical differences between patients with and without muscle wasting.

	Total		Men		Women	
	Muscle wasting (-)	Muscle wasting (+)	Muscle wasting (-)	Muscle wasting (+)	Muscle wasting (-)	Muscle wasting (+)
	n = 63	n = 36	n = 43	n = 20	n = 19	n = 17
Age, years	65.6 (12.9)	73.8 (10.2)***	64.5 (13.9)	71.7 (11.9)*	68.0 (10.0)	76.3 (7.3)**
BMI, kg/m^2^	25.4 (4.0)	22.8 (3.1)**	25.5 (4.3)	21.9 (2.6)**	25.2 (3.4)	23.9 (83.4)
BNP, pg/mL	188.2 (203.4)	560.8 (723.6)***	214.6 (229.8)	686.6 (870.9)**	128.4 (107.1)	412.9 (484.2)*
eGFR, mL/min/1.73 m^2^	64.2 (22.1)	49.9 (28.0)**	63.1(26.4)	44.91(29.5)*	66.8 (16.1)	55.8 (25.6)
TC, μmol/L	62.3 (17.2)	75.2 (46.8)	62.7 (15.4)	88.0 (58.8)	61.2 (21.2)	59.2 (15.9)
FC, μmol/L	48.2 (14.6)	54.4 (31.7)	48.4 (12.8)	62.3(40.1)	47.7 (18.5)	44.7 (11.0)
AC, μmol/L	14.1 (4.6)	20.8 (16.3)	14.3 (4.9)	25.7 (19.7)*	13.5 (4.0)	14.6 (7.6)
AC/FC	0.30 (0.09)	0.38 (0.17)*	0.30 (0.09)	0.42 (0.16)***	0.29 (0.08)	0.34 (0.17)

Data are shown as mean (SD). * p < 0.05, ** p < 0.01, *** p < 0.001. BMI, body mass index; BNP, brain natriuretic peptide; eGFR, estimated glomerular filtration rate; TC, total carnitine; FC, free carnitine; AC, acylcarnitine; AC/FC, the acylcarnitine/free carnitine ratio.

## 4 Discussion

The major findings of the present study are as follows: Plasma BNP and eGFR levels correlated well with the serum AC concentration and the AC/FC. The AC/FC was elevated depending on the stage of CKD. In multivariate analysis, even when corrected for age, sex and BMI, log (eGFR) and log (BNP) were identified as independent factors to define log (serum AC), and log (eGFR) was an independent factor to define log (AC/FC). The serum AC concentration was correlated with LAD, LVDs, and LVEF in men. In addition, the serum AC concentration and AC/FC were negatively correlated with hand-grip strength and anterior thigh MTh in men. The serum AC concentration and the AC/FC were higher in men with versus without muscle wasting. The serum AC concentration and the AC/FC correlated well with several cytokines (TNF-α, GDF-15, and FGF-21).

Carnitine is involved in the transport of FAs in mitochondria mainly in the heart, muscle, and liver by several mechanisms [19]. First, long-chain FAs are converted to acyl-coenzyme A (CoA) esters by ATP-dependent acyl-CoA synthase. Second, these acyl-CoA esters are then converted to AC and free CoA by carnitine palmitoyltransferase (CPT)-I in the mitochondrial outer membrane. The resulting AC is transported across the mitochondrial inner membrane by carnitine-acylcarnitine translocase (CACT). Once within the mitochondrial matrix, the acyl-CoA ester and FC are reformed from AC and free CoA by CPT-II, and FC is exchanged by CACT. In the failing myocardium, dysfunction of these important enzymes may lead to inadequate utilization of substrates, which is reflected by elevated serum levels of long-chain FA intermediate metabolites, such as long-chain AC, in serum [19]. The various AC species that are increased in patients with HF are produced from the corresponding acyl-CoA, and production may occur in one or many tissues, such as heart, muscle, and liver, by several potential mechanisms [20].

In the present study, which examined patients with cardiovascular disease undergoing cardiovascular surgery, the serum AC concentration and the AC/FC were inversely correlated with hand-grip strength and supine anterior thigh MTh in men. AC is a metabolite produced in mitochondria by FA metabolism and might be dysregulated in numerous diseases, affecting muscle [21], [29], [30]. A previous study showed that serum medium- and long-chain AC concentrations were negatively associated with hand-grip strength in linear regression analysis in older men [29]. Also, another study showed that serum short-chain AC concentrations were negatively correlated with hand-grip strength, and short-chain AC levels defined the decline in hand-grip strength at 18 months in community-dwelling elderly subjects [21]. In contrast, there was no relationship between the serum AC concentration and sarcopenia in a study that examined the relationship between carnitine components and nutritional status in patients undergoing gastrointestinal surgery [30]. Thus, further research is needed, because disease and gender may influence the association between serum AC, the AC/FC and muscle function.

Serum AC might contribute to the worsening of HF through the promotion of arrhythmias, insulin resistance, adverse remodeling, and reduced energy production [19]. Previous studies have reported an association between the AC/FC and cardiac events in patients with HF, suggesting that the AC/FC may increase in patients with HF due to mitochondrial dysfunction, which is associated with impaired energy metabolism [13], [14]. Yoshihisa et al. [13] reported that patients with HF with an AC/FC ≥ 0.27 had the highest rate of cardiac events, including cardiac death and HF progression, and the AC/FC was a predictor of cardiac events. Also, Kinugasa et al. [14] found that in patients with HF, sarcopenia was associated with older age (>77 years) and a high AC/FC (>0.31) in patients aged 64–76 years, suggesting that carnitine deficiency is a potential therapeutic target against sarcopenia in patients with HF. The present study showed that the serum AC concentration, but not the AC/FC, is associated with echocardiographic findings and BNP in male patients undergoing cardiac surgery.

The present study showed that the AC/FC, a marker of carnitine deficiency increased with increasing CKD stage. Serum ACs accumulate due to decreased renal clearance of esterified carnitine in patients with chronic renal failure not on HD; patients with CKD are usually reported to have a higher AC/FC [4]. Also, in patients on HD, serum FC levels progressively decrease due to restricted dietary intake, lack of endogenous synthesis in the kidney, and FC removal by HD due to a small molecular weight (161 Da) and its very low protein binding [7], but serum AC levels increase [31]. Yano et al. [31] showed that serum AC levels increased with renal dysfunction independently of urinary excretion levels, while serum FC was not affected by renal function in CKD patients not on HD. These results indicate that AC, unlike FC, is affected by renal function.　Uchiyama et al. [22] also examined the effect of a 6-month home-based exercise program that included aerobic exercise at 40–60 % max heart rate 3 times per week and resistance training at 70 % 1 repetition maximum 2 times per week at home in stage 4 CKD patients (eGFR 15–30 mL/min/1.73 m^2^), and found a greater reduction in AC/FC in the exercise group compared to controls (-0.058 ± 0.024, p = 0.01). The significantly greater reduction in AC/FC in this exercise group suggests improved mitochondrial β-oxidation [13], and mitochondrial dysfunction is not only an important cause of uremic sarcopenia [32], but also is associated with steeper eGFR decline [33]. In addition, improvement of mitochondrial dysfunction may inhibit CKD progression [34].

While the diagnosis of mitochondrial respiratory chain (MRC) dysfunction is hampered by the limited number of surrogates and biomarkers for MRC dysfunction, the hormone-like cytokines, FGF-21 [35], [36] and GDF-15 [37], may have some utility as potential biomarkers for its diagnosis. FGF-21 is a metabolic hormone produced in the liver and expressed in the pancreas and adipocytes [38]; the primary role of FGF-21 is to regulate glucose and lipid metabolism. In mouse skeletal muscle, mitochondrial dysfunction has been reported to induce FGF-21 expression, which may correlate with disease severity and progression in MRC-deficient patients with muscle symptoms [35]. In subsequent studies, FGF-21 was found to be effective in identifying patients with abnormal mitochondrial DNA maintenance [36]. Furthermore, Lehtonen et al. [39] showed that serum FGF-21 can be used as a biomarker for deficient mitochondrial maintenance and translation presenting with muscle symptoms. In contrast, GDF-15 is a member of the transforming growth factor-β cytokine superfamily and plays various roles in cardiovascular disease, inflammation, cancer, and kidney disease [40], [41], [42], [43]. GDF-15 is more sensitive in detecting mitochondrial dysfunction in non-muscle organs; thus, GDF-15 is more sensitive but less specific [44]. Interestingly, a combined evaluation of both GDF-15 and FGF-21 in serum from adult patients with mitochondrial disease did not improve the diagnostic value of the individual tests [45]. Also, a recent study by Riley et al. [46] compared a group of healthy children without mitochondrial disease to a group of children with MRC and suggested that FGF-21 was superior to GDF-15 in discriminating between the two groups.

In the present study, we showed for the first time that both the serum AC concentration and the AC/FC correlate with serum TNF-α, GDF-15, and FGF-21 levels. Several inflammatory cytokines, including TNF-α, are associated with cachexia and anorexia [47], [48]. High levels of TNF-α were also associated with decreased muscle mass and hand-grip strength in the elderly [49]. Furthermore, systemic inflammation and elevated serum TNF-α levels were implicated in various pathologies involving muscle atrophy [50], and GDF-15 was reported to be induced by inflammatory cytokines such as TNF-α [41]. Several previous studies investigated the relationships between serum AC, cytokines such as GDF-15 and FGF-21, and muscle function [51], [52]. Kemp et al. [51] investigated the preoperative and postoperative cross-sectional area (CSA) of the thigh muscle by ultrasound, hand-grip strength, and serum biomarkers, such as AC and GDF-15, in male patients undergoing aortic valve surgery. Preoperative serum AC was inversely correlated with preoperative serum GDF-15 and postoperative muscle CSA and hand-grip strength, suggesting that preoperative mitochondrial dysfunction contributes to postoperative muscle loss, probably due to GDF-15 [51]. Yano et al. [52] followed patients on HD who received either L-carnitine supplementation or exercise therapy for 3 months and showed that L-carnitine supplementation significantly increased muscle mass by BIA and thigh circumference compared with baseline. The change in the serum AC/FC was inversely correlated with the change in thigh circumference before and after L-carnitine administration or exercise, and serum FGF-21 before L-carnitine administration correlated with the change in thigh circumference before and after L-carnitine administration [52]. The present study for the first time found that serum TNF-α, GDF-15, and FGF-21 were potentially associated with serum AC and the AC/FC in cardiac patients undergoing cardiovascular surgery, suggesting that these cytokines might be involved in the association between serum AC and the AC/FC and muscle wasting.

There are several limitations in the present study. Firstly, we recruited patients who underwent kinds of cardiovascular surgery with diverse pharmacological interventions. This might render it challenging to draw definitive conclusions due to the presence of confounding variables. Secondly, we did not have healthy controls. Therefore, further researches including healthy controls should be needed.

## 5 Conclusion

A significant association between the serum AC concentration and the AC/FC ratio with chronic kidney disease and heart failure exists in patients with cardiovascular diseases who undergo cardiovascular surgery. In addition, skeletal muscle loss, muscle wasting, and cardiac remodeling are also linked to the elevation of serum AC and the AC/FC, especially in men. Thus, it is likely that serum AC and the AC/FC are novel biomarkers of chronic kidney disease, cardiac dysfunction, and muscle wasting in patients with cardiovascular diseases who undergo cardiovascular surgery.

## 6 Supporting information captions

This study was supported in part by JSPS KAKENHI Grant Number 19H03981, 22H03457 (to TN). The funding sources for this study had no roles in study design, data collection, analysis, or interpretation. The opinions expressed herein are those of the authors. No additional external funding was received for this study.

## 7 Registration number of clinical studies

The study was approved by the Bioethics Committee of Dokkyo Medical University Hospital (No. 27074), and written informed consent was obtained from all participants.

## CRediT authorship contribution statement

**Takafumi Nakajima:** Writing – original draft. **Taira Fukuda:** Writing – review & editing, Resources, Formal analysis, Conceptualization. **Ikuko Shibasaki:** Resources, Formal analysis. **Syotaro Obi:** Resources, Formal analysis. **Masashi Sakuma:** Resources, Formal analysis. **Shichiro Abe:** Resources, Formal analysis. **Hirotsugu Fukuda:** Supervision. **Shigeru Toyoda:** Supervision, Funding acquisition. **Toshiaki Nakajima:** Writing – review & editing, Supervision, Funding acquisition, Conceptualization.

## Declaration of competing interest

The authors declare that they have no known competing financial interests or personal relationships that could have appeared to influence the work reported in this paper.
